# Association Among *MCT1* rs1049434 Polymorphism, Athlete Status, and Physiological Parameters in Japanese Long-Distance Runners

**DOI:** 10.3390/genes15121627

**Published:** 2024-12-19

**Authors:** Shotaro Seki, Tetsuro Kobayashi, Kenji Beppu, Manabu Nojo, Kosaku Hoshina, Naoki Kikuchi, Takanobu Okamoto, Koichi Nakazato, Inkwan Hwang

**Affiliations:** 1 Faculty of Sport Science, Nippon Sport Science University, 7-1-1, Fukasawa, Setagaya-ku 158-8508, Japan; t-kobayashi@nittai.ac.jp (T.K.); n.kikuchi@nittai.ac.jp (N.K.); tokamoto@nittai.ac.jp (T.O.); hwang@nittai.ac.jp (I.H.); 2 LOGISTEED Track & Field Club House, LOGISTEED, Ltd., Hachigasakimidori-cho, Matsudo-shi 270-0024, Japan; ke.beppu.zo@logisteed.com (K.B.); ma.nojo.fp@logisteed.com (M.N.); 3 Graduate School of Media and Governance, Keio University, 5322, Endo, Fujisawa 252-0882, Japan; kosaku@sfc.keio.ac.jp; 4 Faculty of Medical Science, Nippon Sport Science University, 1221-1, Kamoshida-cho, Aoba-ku, Yokohama 227-0033, Japan; nakazato@nittai.ac.jp

**Keywords:** blood lactate, endurance athletes, aerobic, polymorphism, performance, genetics, *MCT1*

## Abstract

Background/Objectives: Monocarboxylate transporters (MCTs) comprise 14 known isoforms, with MCT1 being particularly important for lactate transport. Variations in lactate metabolism capacity and aerobic performance are associated with the T1470A polymorphism in *MCT1*. We aimed to investigate the frequency of the T1470A polymorphism and compare relevant physiological parameters among long-distance runners, wherein these parameters are fundamental to athletic performance. Methods: We included 158 Japanese long-distance runners (LD) and 649 individuals from the general Japanese population (CON). The frequency of the T1470A polymorphism was compared between these groups and across athletic levels using the chi-square test. Additionally, physiological data were collected from 57 long-distance runners, and respiratory gas measurements were obtained using the mixing-chamber method during a graded incremental exercise test. Results: We observed a significant difference between the LD and CON groups in the dominant model and between the sub-28 min group and 28 min or above group in the recessive model. As the competitive level increased, the frequency of the AA genotype also increased. When comparing physiological parameters between the AA genotype and T allele, subjects with the AA genotype showed significantly higher values for oxygen uptake at lactate threshold (*p* = 0.001), oxygen uptake at onset of blood lactate accumulation (*p* = 0.01), maximal oxygen uptake (*p* = 0.005), and maximal blood lactate concentration (*p* = 0.038). Conclusions: These results suggest that the AA genotype of the T1470A polymorphism of *MCT1* is an effective genotype associated with athletic status and aerobic capacity in Japanese long-distance runners.

## 1. Introduction

Currently, there are 14 known monocarboxylate transporter (MCT) isoforms [1], of which MCT1 is found in various organs throughout the body and is predominantly expressed in slow-twitch muscle fibers [2,3]. MCT1 plays a crucial role in transporting approximately 70–90% of the lactate generated in the body [4] and maintains cellular homeostasis by co-transporting lactate and hydrogen ions in a 1:1 ratio across cell membranes and interstitial spaces [5,6]. Moreover, MCT1, directly and indirectly, facilitates lactate uptake from the bloodstream via MCT4, which is abundant in fast-twitch fibers [7,8,9]. MCT1 content has been reported to vary in response to training intensity, duration, and frequency in humans and animals. For example, in rat skeletal muscle, chronic electrical stimulation leads to an increase in MCT1 content, whereas denervation leads to a decrease [10,11,12]. Additionally, short- and long-term moderate- and high-intensity exercises have been shown to increase MCT1 levels [13]. In human skeletal muscle, MCT1 content increases in response to moderate- to high-intensity training and endurance training, whereas it decreases following detraining [14,15]. MCT1 content is higher in middle- and long-distance runners than in the general population [16], and an increase in MCT1 expression is associated with a shorter time to complete a 10 km cycling test [15]. These findings suggest that an increase in MCT1 content facilitates the efficient uptake of lactate (an energy substrate) into various organs, leading to improved endurance performance.

In recent years, the transport capacity of MCT1 has been reported to vary depending on genetic variations in *MCT1*. The T1470A polymorphism (rs1049434) in *MCT1* (located at 1p12) is a missense mutation in which glutamic acid at codon 490 is replaced by aspartic acid, resulting in a change in the amino acid produced [17]. Merezhinskaya et al. [17] reported that having the AA genotype increased the lactate transport rate by 60–65% compared to having the AT and TT genotypes. Sasaki et al. reported that the variant (A allele) of the T1470A polymorphism exhibited superior lactate transport capacity in mouse skeletal muscle cell lines (C2C12 cells) compared to the wild type (T allele) [18]. Thus, differences in lactate transport capacity across T1470A polymorphisms are currently under study in endurance and power/sprint athletes, as well as in intermittent athletes. Several studies have investigated the relationship between the genotype frequency of the T1470A polymorphism in athletes and their physical abilities. The TT genotype is believed to be prevalent in power and sprint athletes [19,20], whereas the AA genotype is significantly more prevalent in endurance athletes and intermittent sport-specific athletes than in controls [21,22]. In terms of physical abilities corresponding to the T1470A polymorphism, Guilherme et al. (2021) investigated the differences in maximal oxygen uptake (VO_2max_) and found the VO_2max_ of individuals with the AA genotype to be significantly higher than that of those with the TT genotype [23]. Fedotovskaya et al. reported that the maximal blood lactate concentration (BLa_max_) after exercise cessation was significantly lower in subjects with the AA genotype than in those with the AT + TT genotype (T allele) after conducting an incremental test [24]; similar results have been observed in other studies [21,23,25,26]. Based on these findings, the AA genotype exhibits higher VO_2max_ and lactate metabolism capacity compared to the TT genotype and the T allele. Glutamine (A) substitution in the T1470A polymorphism leads to functional activation of MCT1 [27], resulting in more efficient lactate transport and its effective utilization as an energy substrate. This suggests that the AA genotype may be advantageous not only in athletes involved in intermittent sports but also for long-distance runners, where aerobic capacity and lactate metabolism significantly impact performance.

The key factors influencing performance in long-distance runners include VO_2max_, lactate threshold (LT), and running economy [28,29]. Among these, LT, the onset of blood lactate accumulation (OBLA) [30,31,32], and the maximal lactate steady state [33,34] are widely utilized as training intensity and performance indicators in the field of long-distance running coaching. Therefore, a high lactate metabolic capacity is considered one of the essential factors in long-distance runners, as it contributes to efficient high-energy supply during physical activity and helps sustain peak performance. However, research examining the association between the T1470A polymorphism—a genetic factor significantly impacting lactate metabolism—and long-distance runners is limited to a single study by Ben-Zaken et al. [35]. This study only assessed the frequency in a general athletic population, comprising both Ethiopian and non-Ethiopian Israeli individuals [35]. Consequently, the frequency of this polymorphism, including in elite-level athletes, and its association with physiological parameters remain largely unexplored.

This study hypothesized that, among Japanese long-distance runners, the frequency of the AA genotype in the T1470A polymorphism increases with higher competitive levels and that the AA genotype also indicates superior physiological parameters. Therefore, this study aimed to examine and compare the frequency of the T1470A polymorphism with physiological parameters in terms of each genotype among Japanese long-distance runners, including current and former national record holders, Olympic athletes, World Championship participants, Japanese Championship participants, Asian Championship participants, and university-level athletes.

## 2. Materials and Methods

### 2.1. Study Design

We conducted a case–control study to test the hypothesis that the *MCT1* T1470A polymorphism exhibits distinct frequencies across competitive levels. Additionally, we investigated physiological parameters according to the competitive level and the *MCT1* T1470A genotype. Physiological data were obtained using a graded incremental exercise test (GXT), which included both a lactate curve test and a VO_2max_ test. Saliva samples for genetic polymorphism analysis were collected during competitions and training camps. Participants also completed a questionnaire covering age, competitive experience, competitive level, and their 10,000 m personal best record (10,000 m PBR).

### 2.2. Participants

The participants included 158 male Japanese long-distance runners (LD; age: 26.1 ± 9.9 years, competitive experience: 11.9 ± 6.4, mean ± SD) and 649 general Japanese individuals (CON). The CON group consisted of healthy Tokyo residents (271 men, 378 women), while the LD group included former and current Japanese record holders, Olympic athletes, World Championship participants, and long-distance runners with 10,000 m PBR in the 27–30 min range.

The competitive levels within the LD group were categorized based on the Asian 10,000 m PBR benchmarks published by World Athletics, with a cutoff set at sub-28 min vs. 28 min or above. To date, 156 long-distance runners from Asia have achieved sub-28 min for 10,000 m, of whom 139 are Japanese athletes [36]. In 2024, 22 Asian athletes achieved sub-28 min, 20 of whom were Japanese [37]. This suggests that a sub-28 min record represents an elite level among Japanese long-distance runners. The study was approved by the ethics review committee of our institution (023-H120) and was conducted in accordance with the principles outlined in the Declaration of Helsinki. All subjects signed an informed consent form that included (1) the purpose of the study, (2) a statement that the samples would only be used for this study, and (3) explicit anonymity of the final genetic results.

### 2.3. Genotyping

Total DNA was extracted and isolated from 2 mL of saliva collected using a self-collection kit (Oragene^®^ DISCOVER; DNA Genotek, Ottawa, ON, Canada) following the manufacturer’s instructions. The presence of the *MCT1* T1470A polymorphism (rs1049434) was determined using a TaqMan™ SNP genotyping assay (Assay ID: C___2017662_30) and a Real-Time PCR System (CFX96 Touch™ Real-Time PCR, Bio-Rad, Hercules, CA, USA). The FAM dye-labeled probe indicates the genomic A allele, and the VIC indicates the genomic T allele. An increase in FAM or VIC fluorescent signal indicates homozygosity for the FAM- or VIC-specific allele, while an increase in both signals indicates heterozygosity. A scatter plot showing the endpoint fluorescence signals was used to discriminate the genotypes. We performed each PCR reaction in a 6 μL genotyping mixture containing 2.5 μL TaqMan Universal Master Mix II, 0.125 μL TaqMan SNP Genotyping Assay mix, 2.375 μL sterilized water, and 1 μL genomic DNA. The genotyping results were analyzed using the CFX Manager software version 2.1 (Bio-Rad, Hercules, CA, USA).

### 2.4. GXT

For collection of physiological parameter data, GXT was conducted with 57 long-distance runners. The lactate curve test began with a 3 min warm-up at a speed of 200 m/min. Afterward, participants wore a mask (Hans Rudolf Inc., Shawnee, KS, USA), and physiological parameters were measured using a pulmonary exercise load monitoring system (AE 310S, Minato Medical Science Co., Ltd., Kanagawa, Japan) with the mixing-chamber method. Heart rate was measured using a heart rate sensor (H10, Polar Inc, Kainuu, Finland). Next, the measurement with the heart rate sensor and pulmonary exercise load monitoring system was synchronized. After measuring 1 min of rest, the first speed was set to 220 m/min for 3 min of steady-state running, followed by a 1 min rest period. During the 1 min rest period, subjective exertion was assessed using the Rating of Perceived Exertion (RPE) scale, and blood lactate was measured using a Lactate Pro 2 (Arkray Inc, Kyoto, Japan). Speed was increased incrementally by 20 m/min per stage until blood lactate concentration exceeded 4 mmol/L. Thereafter, a 2 min rest was allowed before beginning the VO_2max_ test. The VO_2max_ test began at a speed of one stage below the point at which lactate concentration exceeded 4 mmol/L. The speed was then increased by 10 m/min every 30 s until the runner reached a pace that could be maintained for 1–1.5 min, after which the test continued until exhaustion (all-out). Blood lactate was measured immediately after the test, as well as at 3 min and 5 min after the test, using fingertip blood samples. Finally, a cool-down was performed on a cycle ergometer or the track.

The criterion for determining VO_2max_ was defined as the highest oxygen uptake recorded every 30 s. Additionally, at least three of the following conditions needed to be met: (1) leveling-off of oxygen uptake, (2) measured heart rate reaching the maximal heart rate [38], (3) respiratory exchange ratio of 1.15 or higher, (4) RPE of 18 or above, and (5) blood lactate concentration of 8 mmol/L or higher following exercise [39].

The determination of VO_2_-LT was based on the definition by Beaver et al. [40]. It involved calculating the slopes of the linear portions of the lactate curve above and below the inflection point in terms of blood lactate concentration and identifying the intersection of these two lines as the VO_2_-LT. For the determination of VO_2_-OBLA, the oxygen uptake corresponding to a blood lactate concentration of 4 mmol/L was used.

The BLa_max_ was defined as the highest value that was obtained during post-exercise measurement of blood lactate.

### 2.5. Statistical Analysis

The Hardy—Weinberg equilibrium of the observed genotype frequencies was assessed using the χ^2^ test and Fisher’s exact probability test. Additionally, the differences in genotype frequencies (AA, TA, and TT) of the rs1049434 polymorphism between the LD and CON groups were tested. Comparisons were also made between the LD and CON groups, between different competition levels and the CON group, and between the competition levels themselves. To estimate the contribution of the *MCT1* T1470A polymorphism, odds ratios (ORs) and 95% confidence intervals (CIs) were calculated. Three genetic models were used: the codominant model (AA vs. AT or TT), the dominant model (AA + AT vs. TT), and the recessive model (AA vs. TT + AT).

Physiological parameters associated with the *MCT1* T1470A genotypes are presented as mean ± standard deviation. The normality of the data was assessed using the Shapiro—Wilk test. If normality was confirmed, a parametric independent *t*-test was performed, and *p*-values and 95%CI were reported. The effect size of the *t*-test was evaluated using Cohen’s d. The classification of effect size was as follows: small (0.2–0.6), medium (0.6–1.2), large (1.2–2.0), and very large (≥2.0) [41]. The 95%CI for the effect size was also presented. If normality was not confirmed, a non-parametric Mann–Whitney U-test was performed. The *p*-value, 95%CI, and effect size were reported. The effect size was evaluated using r, and the classification of effect size was as follows: small (0.1–0.3), medium (0.3–0.5), and large (≥0.5) [42].

The χ^2^ test and Fisher’s exact probability test were conducted using R Studio Statistical Software (V 4.4.1). The independent *t*-test and Mann–Whitney U-test were performed using SPSS Version 29.0 (IBM Corp., Armonk, NY, USA) for statistical analysis. The statistical significance level was set at *p* < 0.05.

## 3. Results

### 3.1. Characteristics of Japanese Long-Distance Runners

The characteristics of Japanese long-distance runners categorized by the T1470A polymorphism, along with their personal best records for the 10,000 m event, are summarized in Table 1.

### 3.2. The Distribution of T1470A Polymorphism in LD and CON Groups

The distribution of the T1470A polymorphism frequency was examined in the LD and CON groups. In the CON group, the frequencies of the AA, AT, and TT genotypes were 47.0%, 42.7%, and 10.3%, respectively, which conformed to a Hardy—Weinberg equilibrium (CON: *χ*^2^ = 0.12, *p* = 0.941; Table 2). In contrast, the values in the LD group were 41.8%, 53.2%, and 5.1%, respectively, which were not in a Hardy—Weinberg equilibrium (*χ*^2^ = 8.28, *p* = 0.016; Table 2). The allele distributions of LD and CON groups are shown in Table 2.

### 3.3. Comparison of Genetic Model Frequencies Between LD and CON Groups

The genetic model frequencies of the T1470A polymorphism for the LD and CON groups in this study are shown in Table 3. No significant differences were observed in the codominant and recessive models. However, a significant difference was found in the dominant model between the LD and CON groups (OR: 2.16, 95%CI: 1.01–4.59, *p* = 0.04).

### 3.4. Comparison of Genetic Model Frequencies Between Athletic Levels in the LD and CON Groups

The genetic model frequencies for the T1470A polymorphism among groups with 10,000 m PBR sub-28 min and 28 min or above, as well as in the CON group, are presented in Table 4. No significant differences were observed in the frequencies of any models between the sub-28 min group and the CON group. However, between the 28 min or above group and the CON group, a significant difference was noted for the AT genotype (OR: 1.58, 95%CI: 1.08–2.31, *p* = 0.02), although there was no significant difference for the AA or TT genotype. Additionally, no significant differences were observed in either the recessive or dominant model (OR: 0.72, 95%CI: 0.50–1.05, *p* = 0.08; OR: 2.20, 95%CI: 0.99–4.91, *p* = 0.05, respectively).

Comparing the sub-28 min group with the 28 min or above group, no significant differences were found for the AA and TT genotypes. However, a significant difference was observed for the AT genotype (OR: 0.32, 95%CI: 0.10–0.96, *p* = 0.03). Furthermore, significant differences were found in the recessive model (OR: 2.87, 95% CI: 1.00–8.19, *p* = 0.04), while no significant difference was observed in the dominant model (OR: 0.84, 95%IC: 0.10–7.24, *p* = 1.00).

### 3.5. Comparison of Aerobic Capacity Between the AA Genotype and T Allele

**Figure 1:**
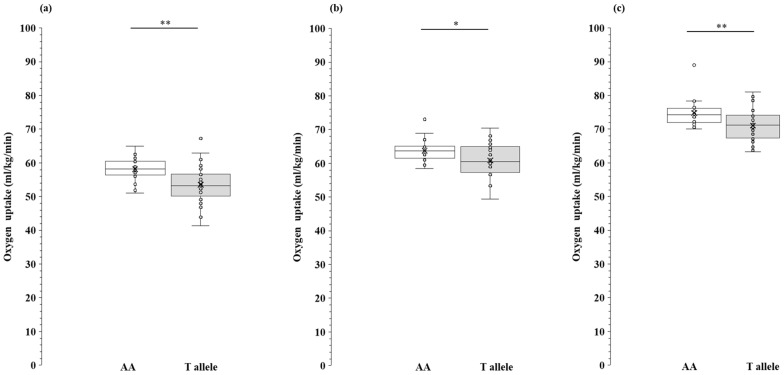
Comparison of aerobic capacity between MCT1 AA genotype and T allele in long-distance runners. VO_2_-LT and VO_2_-OBLA were analyzed using independent *t*-test. VO_2max_ was analyzed using the Mann–Whitney U-test. Box whisker plots (line: median; cross: mean; box: data from first to third quartile; whisker: ±1.5 × interquartile range) with individual plots (circle). (**a**) VO_2_-LT, Oxygen uptake at lactate threshold (relative value); (**b**) VO_2_-OBLA, Oxygen uptake at onset of blood lactate accumulation (relative value); (**c**) VO_2max_, Maximal oxygen uptake (relative value); AA, AA genotype (blank box whisker plot); T allele, TT genotype + AT genotype (gray box whisker plot). *: *p* < 0.05; **: *p* < 0.01.

The results in Figure 1 indicate that the VO_2_-LT, VO_2_-OBLA, and VO_2max_ for the AA genotype carriers were significantly higher than those for the T-allele carriers (*p* = 0.001, 95% CI: 1.91–7.18, d = 0.948; *p* = 0.01, 95% CI: 0.72–5.09, d = 0.664; *p* = 0.005, 95% CI: 1.20–5.80, r = 0.37; see Supplementary Tables S1 and S2).

### 3.6. Comparison of Maximal Blood Lactate Concentration Between the AA Genotype and T Allele

**Figure 2:**
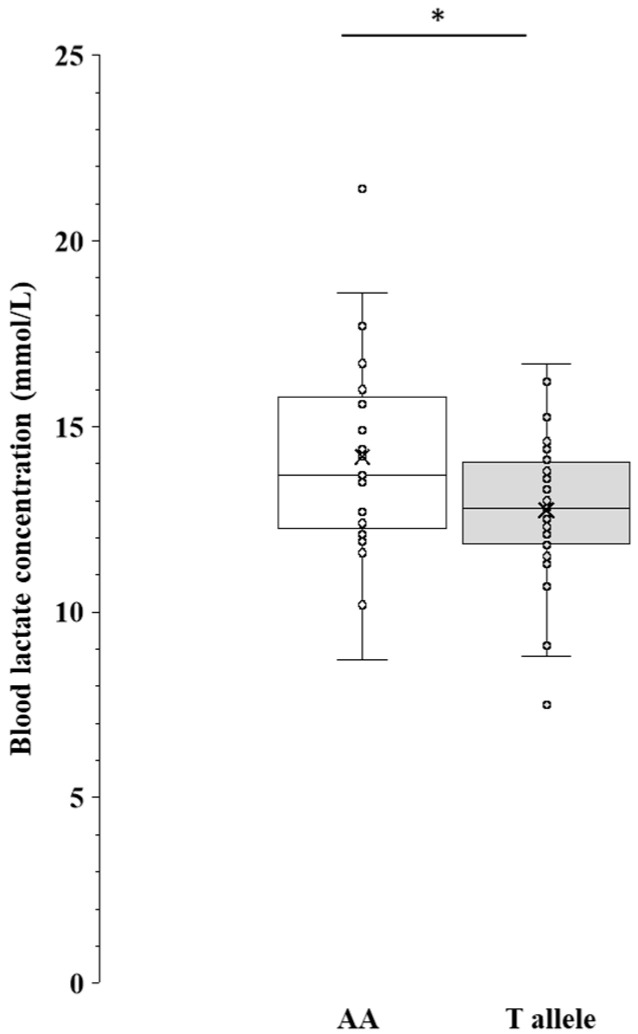
Comparison of the maximal blood lactate concentration after GXT between MCT1 AA genotype and T allele in long-distance runners. BLa_max_ was analyzed using independent *t*-test. Box whisker plots (line: median; cross: mean; box: data from first to third quartile; whisker: ±1.5 × interquartile range) with individual plots (circle). BLa_max_, the maximal blood lactate concentration; AA, AA genotype (blank box whisker plot); T allele, TT genotype + AT genotype (gray box whisker plot). *: *p* < 0.05.

A comparison of the BLa_max_ after GXT is shown in Figure 2. The BLa_max_ was significantly higher in the AA genotype carriers than in the T-allele carriers (*p* = 0.038, 95% CI: 0.08–2.77, d = 0.585; see Supplementary Table S1).

## 4. Discussion

This study used a case–control design to examine the frequency of the T1470A polymorphism and compare physiological parameters in Japanese long-distance runners. The main findings of this study revealed that, as the competitive level increased, the frequency of the AA genotype also increased among Japanese long-distance runners. Additionally, in terms of physiological parameters, the AA genotype, which is considered to confer superior lactate metabolism ability, showed a significantly better association with physiological parameters than the T allele. These findings suggest that the AA genotype of the T1470A polymorphism is associated with a higher status and physical performance in Japanese long-distance runners.

The genotype frequency of the T1470A polymorphism in Japanese elite long-distance runners in this study deviated from the Hardy–Weinberg equilibrium (HWE). Several factors, including methodological considerations and conditions not aligning with the equilibrium of allele transmission, could contribute to this deviation [43]. However, the analysis methods employed in this study are established and validated [21,44], suggesting that methodological issues are unlikely to have influenced the results. Additionally, the *MCT1* gene, involved in mitochondrial aerobic metabolism [45], may not follow strict Mendelian inheritance patterns [46], which could explain the deviation from HWE observed in this study. This deviation has also been observed in other studies focusing on elite athletes, indicating that such findings are not unique to this study [47,48].

To date, the only study investigating the frequency of the T1470A polymorphism in long-distance runners is the one by Ben-Zaken et al. [35]. In this study, 113 Israeli long-distance runners (37 of Ethiopian origin [33 men, 4 women], 76 of non-Ethiopian origin [59 men, 17 women]) and 55 controls were analyzed for differences in the genotype and genetic models of the T1470A polymorphism. The results showed that among Ethiopian-origin long-distance runners, the AA, AT, and TT genotype frequencies were 65%, 35%, and 0%, respectively; for non-Ethiopian-origin runners, they were 45%, 42%, and 13%; and for the control group, they were 42%, 49%, and 9%, respectively. The Ethiopian-origin athletes, who were at a higher competitive level, exhibited a significantly higher frequency of the AA genotype than both the non-Ethiopian-origin athletes and the control group. Furthermore, the frequency of the AA genotype + the AT genotype (A allele) in Ethiopian-origin athletes was 82%, which was higher than the 66% observed in the non-Ethiopian-origin and control groups. Guilherme et al. [23] investigated the frequency of the T1470A polymorphism in a study involving 1208 Brazilians (319 endurance athletes [238 males, 81 females] and 890 general population [506 males, 384 females]) and 867 Europeans (382 Russians [208 endurance athletes, 174 general population] and 485 Hungarians [107 endurance athletes, 378 general population]). The study reported that in Brazilian white endurance athletes, international-level athletes showed a lower frequency of the TT genotype than domestic-level and regional-level athletes (TT genotype: international 14.5% vs. domestic 19.4% vs. regional 22%). Conversely, the frequency of the A allele was higher in international-level athletes than in domestic- and regional-level athletes (A allele: international 63% vs. domestic 59% vs. regional 57%). Furthermore, the study found that in Europeans, the frequency of the A allele was higher in endurance athletes than in the general population [23]. Our study showed that, in the overall group of Japanese long-distance runners, the frequency of the A allele was higher, and the frequency of the TT genotype was lower than those in the control group. Furthermore, as the competitive level increased, the frequency of the AA genotype was found to be higher. These findings are consistent with the results of previous studies. Taken together, the characteristic feature of the T1470A polymorphism in Japanese long-distance runners suggests that, overall, the frequency of the A allele tends to be higher, and as the competitive level increases, the expression of the AA genotype becomes more pronounced.

In this study, we first examined the relationship between Japanese long-distance runners and the T1470A polymorphism. The results revealed that as the competitive level increased, the frequency of the AA genotype increased, indicating that the AA genotype plays a crucial role in reaching a highly competitive level. Therefore, our study also simultaneously investigated the differences in physiological parameters between the AA genotype and T allele in Japanese long-distance runners. Although there are few previous studies on this topic, several studies have examined the physiological responses to different polymorphisms, including in the general population and among various endurance athletes. Gasser et al. [45] conducted a study on 22 healthy general British individuals using a cycle ergometer to examine VO_2max_. The results showed a tendency of the AA genotype carriers to have higher values (54.4 ± 3.0 mL/kg/min) than the T-allele carriers (51.3 ± 1.8 mL/kg/min), but no significant difference was found between the two genotypes [45]. Similarly, Pasqualetti et al. [49] investigated the differences in physical performance by T1470A genotypes in 27 elite rugby players. They found no significant difference between the AA genotype (49.1 ± 3.5 mL/kg/min) and the T allele (48.4 ± 4.6 mL/kg/min) [49]. On the other hand, Guilherme et al. examined the differences in VO_2max_ by genotypes in 46 Russian endurance athletes and reported that the AA genotype carriers showed significantly higher values than the TT genotype carriers [23]. Therefore, the results obtained in this study support the findings of Guilherme et al. [23]. We examined physiological parameters related to aerobic capacity, including VO_2max_, which is a representative parameter of aerobic ability, as well as VO_2_-LT and VO_2_-OBLA, which have not been reported in previous studies. The results showed that these parameters of aerobic capacity in the AA genotype carriers were significantly higher than those in the T-allele carriers. Gasser et al. [45] investigated the muscle fiber composition characteristics associated with the T1470A genotype in the general population. They found that the AA genotype carriers had a higher percentage of type I fibers than the T-allele carriers (AA: 47.8 ± 4.1% vs. T allele: 38.2 ± 1.9%), and the proportion of muscle fibers occupied by type I fibers was also greater for the AA genotype (AA: 44.2 ± 3.1% vs. T allele: 32.2 ± 2.6%). This is the only study that has approached the biochemical aspects of the T1470A polymorphism in this context. Based on these findings, it is possible that the results are influenced by the muscle fiber composition characteristics associated with the T1470A polymorphism. It has been shown that long-distance runners have a higher percentage of type I muscle fibers than the general population and explosive/power athletes [50,51], and these muscle fibers are related to VO_2max_ and VO_2_-LT [52,53]. Additionally, increases in VO_2_-LT and VO_2_-OBLA are associated with a reduction in lactate production or an increase in lactate clearance, or a combination of both, during physical activity [54,55]. The lack of significant differences in VO_2max_ between T1470A genotypes in previous studies may be due to factors such as the lack of a clear classification of athletes’ sports disciplines, which could have led to either overestimation or underestimation. Based on our study, although we did not assess muscle fiber proportions, the AA genotype in long-distance runners may potentially be associated with a higher aerobic capacity than the T allele. Therefore, it is inferred that athletes with the AA genotype may demonstrate superior oxygen utilization, oxygen delivery, and lactate metabolism in both the whole body and skeletal muscles.

The BLa_max_ was higher in the AA genotype carriers than in the T-allele carriers, which is inconsistent with previous studies. Fedotovskaya et al. [24] examined post-exercise blood lactate concentrations immediately after a graded rowing test in 79 rowers and found that the AA genotype was associated with lower levels of lactate concentration than the T allele. Similarly, Guilherme et al. [23] reported that endurance athletes with the AA genotype, including rowers, canoeists, and triathletes, had lower post-exercise BLa_max_ than those with the TT genotype. Additionally, Kikuchi et al. [21] examined blood lactate levels during a 10 s sprint interval test in elite Japanese wrestlers and found that at the fifth sprint and immediately after the test, the AA genotype carriers exhibited lower blood lactate concentrations than the T-allele carriers. Massidda et al. [26] investigated the performance and blood lactate concentrations during a repeated sprint test (30 m × 6 sprints) in soccer players with different T1470A genotypes. They found that those with the AA genotype and A allele exhibited significantly lower average times to complete the fifth sprint and showed superior performance on the sixth sprint compared to those with the TT genotype. Moreover, while the differences in blood lactate concentrations during the sprints were unclear, the study reported that post-exercise blood lactate concentrations immediately after the test and 3 min afterward were lower in individuals with the A allele than in those with the TT genotype. Hui et al. [56] suggested that lactate is a primary metabolic substrate for energy production in the aerobic energy supply pathway, specifically within the TCA cycle. They reported that during moderate- to high-intensity exercise, the metabolic turnover of glucose in all organs and cells increases approximately 2.3 times compared to values observed during rest, while the metabolic turnover of lactate increases by about 4.5 times [56]. According to these studies, it is hypothesized that subjects with the AA genotype, which is associated with a higher proportion of slow-twitch muscle fibers, likely exhibit superior oxygen utilization and oxygen transport capacity in muscle tissue during physical activity. As a result, lactate is efficiently metabolized as an energy substrate within the muscles and bloodstream, contributing to sustained high performance during prolonged exercise. Gasser et al. [45] reported that the AA genotype is associated with efficient aerobic energy supply using glucose or glycogen. This suggests that the AA genotype is well suited for rapid energy production through carbohydrate metabolism, and it is also associated with a high metabolic capacity to handle the increased lactate production that accompanies this energy supply. This high metabolic capability is thought to contribute to the ability to sustain high performance over extended periods. Furthermore, Higuchi et al. [27] found that the glutamate (A) substitution in the T1470A polymorphism leads to a conformational change in *MCT1*, which is involved in the functional activation of the MCT1 protein [27]. According to Thomas et al. [57], the protein content of MCT1 and MCT4, both of which are involved in the influx and efflux of lactate in tissues, increases with training. However, MCT1 content is about twice as high as that of MCT4 in terms of the ratio within tissues, indicating that MCT1 is particularly sensitive to training effects. Moreover, Bonen et al., based on the findings of McCullagh et al. and unpublished data from Bonen, reported that an increase in MCT1 protein content alone is associated with enhanced lactate influx and efflux [13,58,59]. Therefore, long-distance runners with the AA genotype, who have higher levels of the MCT1 protein and enhanced functional activity than the general population, may not only have a greater ability to uptake lactate into tissues but also a higher capacity to export lactate from tissues to the bloodstream than individuals with the T allele. Hence, it is possible that the higher blood lactate concentrations observed after maximal exercise in this study are due to the superior lactate transport ability of athletes with the AA genotype.

Our study has some limitations that warrant further consideration. First, the physiological parameters were measured only in runners who voluntarily agreed to participate in the study. As a result, the selection of participants may have introduced a potential bias. Second, the physiological parameters observed among the genetic polymorphisms in this study do not necessarily reflect the outcomes of the entire population in the case–control design. In future studies, comprehensive data collection on both athletic status and physiological capacity, wherever possible, is necessary. Moreover, further research should investigate the relationship between physical performance and physiological parameters in combination with the 251 sport-related genetic polymorphisms, which have already been associated with athlete status and physical performance. By examining these genetic polymorphisms, it will be possible to identify the specific genetic factors required for each sport, shedding light on the genetic determinants of athletic performance.

These findings suggested that the AA genotype of the T1470A polymorphism is associated with higher athletic status and physical capabilities in Japanese long-distance runners.

## 5. Conclusions

This study investigated the frequency of the *MCT1* T1470A polymorphism and compared physiological parameters among Japanese long-distance runners, including former and current national record holders, Olympians, and World Championship participants with a 10,000 m PBR of 27–30 min, as well as general Japanese individuals. The findings revealed that, overall, the A allele was more prevalent, and the TT genotype was less common, among Japanese long-distance runners. Additionally, as the competitive level increased, the frequency of the AA genotype was the highest. Furthermore, physiological parameters, such as VO_2max_, VO_2_-LT, and VO_2_-OBLA, as well as post-exercise BLa_max_, were significantly higher for the AA genotype than for the T allele.

Based on these results, it is suggested that the AA genotype of the *MCT1* T1470A polymorphism is an effective genotype associated with athletic status and aerobic capacity in Japanese long-distance runners.

## Figures and Tables

**Table 1:** Characteristics of Japanese long-distance runners and 10,000 m PBR.

*MCT1* rs1049434	n (158)	Age (yrs)			Career (yrs)			10,000 m PBR (MM:SS)		
AA	66	26.7	±	10.2	12.6	±	6.4	29:07	±	1:35
AT	84	25.8	±	9.6	11.7	±	6.5	29:04	±	1:10
TT	8	24.5	±	11.1	9.5	±	5.6	30:03	±	1:59
A allele	150	26.1	±	9.9	12.1	±	6.4	29:05	±	1:21
T allele	92	25.6	±	9.7	11.5	±	6.4	29:08	±	1:15

Data are presented as mean ± SD. 10,000 PBR, 10,000 m personal best record; A allele, AA genotype + AT genotype; T allele, AT genotype + TT genotype.

**Table 2:** Genotype and allele frequencies of *MCT1* T1470A polymorphism in the LD and CON groups.

		n	Genotype Frequency, % (n)			HWE *p*-Value	Allele, %	
			AA	AT	TT		A	T
*MCT1* T1470A polymorphism								
	LD	158	41.8 (66)	53.2 (84)	5.1 (8)	0.016 *	68.4	31.6
	CON	649	47.0 (305)	42.7 (277)	10.3 (67)	0.941	68.3	31.7

Data were analyzed using Hardy–Weinberg equilibrium (HWE). LD, long-distance runners; CON, controls. *: *p* < 0.05.

**Table 3:** Comparison of the frequencies of the codominant, recessive, and dominant models between LD and CON groups.

Genetic Model	CON % (n)	LD % (n)	CON vs. LD		
			*p*-Value	OR	95%CI
Codominant					
AA	47.0 (305)	41.8 (66)		1.00	
AT	42.7 (277)	53.2 (84)	0.07	1.40	0.98–2.01
TT	10.3 (67)	5.1 (8)	0.13	0.55	0.25–1.20
Recessive					
AA	47.0 (305)	41.8 (66)	0.24	0.89	0.57–1.15
AT + TT	53.0 (344)	58.2 (92)		1.00	
Dominant					
AA + AT	89.7 (582)	94.9 (150)	0.04 *	2.16	1.01–4.59
TT	10.3 (67)	5.1 (8)		1.00	

Data were analyzed using chi-square test. Recessive: AA genotype vs. TT genotype + AT genotype; Dominant: AA genotype + AT genotype vs. TT genotype; Codominant: AA genotype vs. AT genotype or TT genotype; LD, long-distance runners; CON, controls; OR, odds ratio; 95%CI, 95% confidence interval. *: *p* < 0.05.

**Table 4:** Comparison of the frequencies of the codominant, recessive, and dominant models between athletic levels in the LD and CON groups.

Genetic Model	CON % (n)	Sub-28 min % (n)	28 min or Above % (n)	CON vs. Sub-28 min			CON vs. 28 min or Above			28 min or Above vs. Sub-28 min		
				*p*-Value	OR	95%CI	*p*-Value	OR	95%CI	*p*-Value	OR	95%CI
Codominant												
AA	47.0 (305)	64.7 (11)	39.0 (55)		1.00			1.00			1.00	
AT	42.7 (277)	29.4 (5)	56.0 (79)	0.22	0.50	0.17–1.46	0.02 *	1.58	1.08–2.31	0.03 *	0.32	0.10–0.96
TT	10.3 (67)	5.9 (1)	5.0 (7)	0.70	0.41	0.05–3.26	0.19	0.57	0.25–1.33	1.00	0.71	0.08–6.40
Recessive												
AA	47.0 (305)	64.7 (11)	39.0 (55)	0.15	2.07	0.76–5.66	0.08	0.72	0.50–1.05	0.04 *	2.87	1.00–8.19
AT + TT	53.0 (344)	35.3 (6)	61.0 (86)		1.00			1.00			1.00	
Dominant												
AA + AT	89.7 (582)	94.1 (16)	95.0 (134)	1.00	1.84	0.24–14.11	0.05	2.20	0.99–4.91	1.00	0.84	0.10–7.24
TT	10.3 (67)	5.9 (1)	5.0 (7)		1.00			1.00			1.00	

Data were analyzed using chi-square test. Recessive: AA genotype vs. TT genotype + AT genotype; Dominant: AA genotype + AT genotype vs. TT genotype; Codominant: AA genotype vs. AT genotype or TT genotype; sub-28 min, less than 28 min; 28 min or above, 28 min or more; CON, controls; OR, odds ratio; 95%CI, 95% confidence interval. *: *p* < 0.05.

## Data Availability

The data presented in this study are available from the corresponding author due to their sensitive nature. As the dataset contains privacy-sensitive details, we are unable to make it publicly accessible in an open-access format.

## Supplementary Materials

The following supporting information can be downloaded at: https://www.mdpi.com/article/10.3390/genes15121627/s1, Table S1: Comparison of physiological parameters between AA genotype and T allele in Japanese long-distance runners; Table S2: Comparison of physiological parameters between AA genotype and T allele in Japanese long-distance runners.
